# Supplementary material for: Hereditary variants of unknown significance in African American women with breast cancer
Source: PLoS One. 2022 Oct 31;17(10):e0273835. doi: 10.1371/journal.pone.0273835 (PMC9621418; doi:10.1371/journal.pone.0273835)
Supplement: Attachment — Submitted filename: Response to Reviewers 8_12_2022.docx [file pone.0273835.s004.docx]

We would like to thank the reviewers for their comments. Below, we have responded (in red) to each of the concerns raised.

Reviewer #1: The authors have addressed all of my previous concerns. There are only 2 minor points to address.
1. In Table 1, the authors should replace SNP with missense. SNP suggests that the change is a benign polymorphism whereas missense more accurately describes the type of change.

The change has been made.

2. the dbGAP accession number should be included in the manuscript.

The accession number (phs002977) was added at line 112 of the document with no track changes.
